# StarScan: a web server for scanning small RNA targets from degradome sequencing data

**DOI:** 10.1093/nar/gkv524

**Published:** 2015-05-18

**Authors:** Shun Liu, Jun-Hao Li, Jie Wu, Ke-Ren Zhou, Hui Zhou, Jian-Hua Yang, Liang-Hu Qu

**Affiliations:** 1 Key Laboratory of Gene Engineering of the Ministry of Education, Sun Yat-sen University, Guangzhou 510275, P. R. China; 2 State Key Laboratory for Biocontrol, Sun Yat-sen University, Guangzhou 510275, P. R. China

## Abstract

Endogenous small non-coding RNAs (sRNAs), including microRNAs, PIWI-interacting RNAs and small interfering RNAs, play important gene regulatory roles in animals and plants by pairing to the protein-coding and non-coding transcripts. However, computationally assigning these various sRNAs to their regulatory target genes remains technically challenging. Recently, a high-throughput degradome sequencing method was applied to identify biologically relevant sRNA cleavage sites. In this study, an integrated web-based tool, StarScan (sRNA target Scan), was developed for scanning sRNA targets using degradome sequencing data from 20 species. Given a sRNA sequence from plants or animals, our web server performs an ultrafast and exhaustive search for potential sRNA–target interactions in annotated and unannotated genomic regions. The interactions between small RNAs and target transcripts were further evaluated using a novel tool, alignScore. A novel tool, degradomeBinomTest, was developed to quantify the abundance of degradome fragments located at the 9–11th nucleotide from the sRNA 5′ end. This is the first web server for discovering potential sRNA-mediated RNA cleavage events in plants and animals, which affords mechanistic insights into the regulatory roles of sRNAs. The StarScan web server is available at http://mirlab.sysu.edu.cn/starscan/.

## INTRODUCTION

Eukaryotic genomes encode thousands of small non-coding RNAs (sRNAs), such as microRNAs (miRNAs), PIWI-interacting RNAs (piRNAs) and endogenous small interfering RNAs (endo-siRNAs) (1–5). By base pairing to mRNAs, these sRNAs downregulate their targets through two distinct modes, either Argonaute-catalyzed cleavage or a second mode that involves mRNA destabilization and translational repression (1–5). These RNA molecules are key regulators of diverse cellular processes, including proliferation, apoptosis, differentiation and the cell cycle (1–6).

Argonaute-catalyzed cleavage often requires extensive complementarity between a small RNA and its target (1–6). Such direct cleavage of RNAs is often guided by piRNAs, endo-siRNAs and plant miRNAs (1–6). Extensive pairing between these RNAs and their targets allows for direct RNA-induced silencing complex mediated cleavage of the mRNA at nucleotides 9–11 of the paired sRNAs (1–6). Although the majority of animal miRNAs destabilize mRNAs by imperfect base pairing to targets, few animal miRNAs can mediate cleavage of target RNAs with a highly complementary interaction. For example, direct cleavage of endogenous HOXB8 mRNA guided by miR-196 has been reported in human cells (7). In addition, several examples of direct cleavage of mRNAs were identified from degradome sequencing data in human and mouse cells (8,9). Surprisingly, a recent study reported that cnidarian miRNAs frequently regulate targets by cleavage (10). Despite these intriguing studies of individual miRNA-mediated cleavage of mRNAs in animals, it is still unclear how many animal miRNAs can regulate mRNAs via cleavage.

Although many studies that address small RNAs have focused on defining their protein-coding gene regulatory functions, increasing numbers of researchers are assigning them to novel classes of regulatory target genes, including multiple classes of ncRNAs (lncRNAs, circRNAs and pseudogene-derived lncRNAs) (1–6). For example, miR-671 directs the cleavage of a circular antisense transcript (CDR1AS) of the Cerebellar Degeneration-Related protein 1 (CDR1) locus in an Ago2-slicer-dependent manner (11). Recent studies showed that the degradation of spermatogenic cell-specific lncRNAs was mediated by piRNAs (12,13). However, since eukaryotic genomes encode thousands of lncRNAs, circRNAs and pseudogene-derived lncRNAs, predicting the interactions between these ncRNAs and thousands of sRNAs remains a daunting challenge.

In recent years, significant efforts have been made in determining biologically relevant sRNA–target interactions using high-throughput experimental approaches. Parallel analysis of RNA ends (PARE) (14,15) and genome-wide mapping of uncapped and cleaved transcripts (GMUCT) (16) approaches have been developed to discover genuine sRNA-mediated RNA cleavage events in multiple plant (17,18) and animal species (8,9). However, most degradome sequencing studies have focused on protein-coding targets. In fact, our genome-wide target plots (t-plots) have revealed that there are plenty of significant cleavage signals located in non-coding transcripts or unannotated regions (19). This type of data is a rich trove that is worthy of mining for novel targets of the known and novel sRNAs.

While several stand-alone tools, including CleaveLand (20), SeqTar (21), sPARTA (22) and PAREsnip (23), were developed to predict cleaved sRNA targets from degradome sequencing data, they focused on plant miRNAs and have not yet been used for animal sRNAs. With the exception of our StarBase platform (19) and the latest tool called sPARTA (22), these software exclusively search sRNA targets from annotated, not unannotated, transcripts. Moreover, although thousands of piRNAs have been identified from next-generation sequencing data, no software has been developed to predict their cleavage targets from degradome sequencing data. Therefore, with the increasing amount of degradome sequencing data available, there is a substantial need to develop a web-based tool to integrate these large-scale data and explore the interactions between diverse sRNAs and multiple classes of genes.

**Figure 1.**
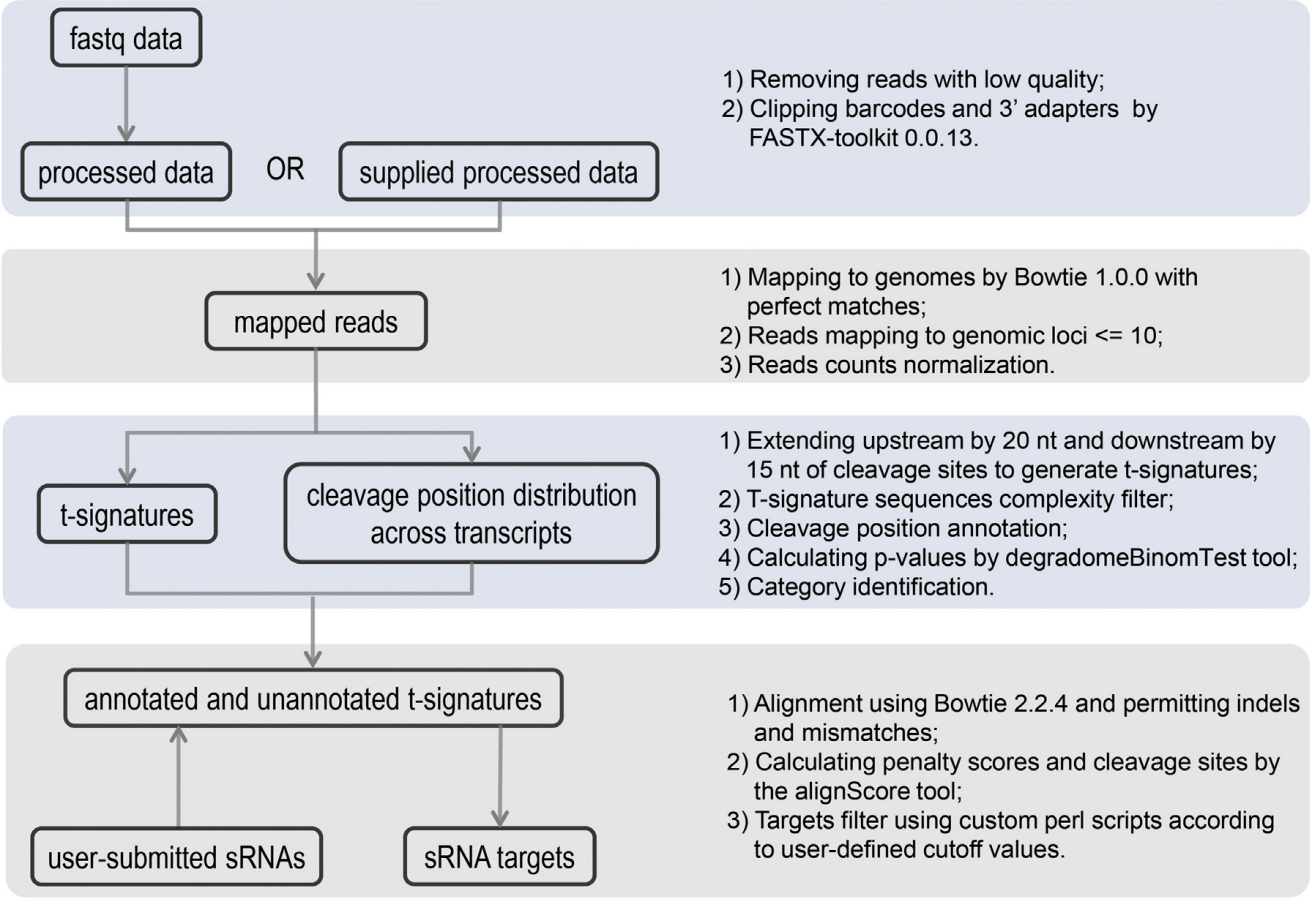
The sRNA target analysis workflow of StarScan. The step description is listed at the right of the boxes.

Here, we present StarScan (sRNA target Scan), the first public server for scanning sRNA targets utilizing degradome sequencing data from 20 species (Figure 1). In addition to being able to analyze plant miRNA targets as with existing tools, our web server can predict targets of diverse animal sRNAs including miRNAs, piRNAs and endo-siRNAs. StarScan was also designed to search for small RNA targets in annotated and unannotated genomic regions. We believe that this web server will help researchers investigate new sRNA–target interactions and discover novel regulatory modules from degradome sequencing data.

## StarScan ANALYSIS WORKFLOW

### Data sources and gene annotation

Degradome sequencing data, namely PARE and GMUCT datasets, were retrieved from the Gene Expression Omnibus (GEO) and Sequence Read Archive (SRA) (24). One hundred samples from 33 individual studies involving 20 species were integrated into StarScan. All reference genome sequences and gene annotations of plants were downloaded from the Ensembl Plants Database (25). *Caenorhabditis elegans* and *Nematostella vectensis* genome sequences and gene annotations were downloaded from the Ensembl Metazoa database (25). The reference genome sequences and gene annotations of human and mouse were downloaded from UCSC bioinformatics web sites (26) and GENCODE (27), respectively.

### Identifying genome-wide cleavage signatures from degradome sequencing data

Barcodes or 3′-adapters of raw degradome sequencing data were clipped using the FASTX-toolkit software (version 0.0.13). The low-complexity reads that only contained 1 or 2 unique nucleotides were then discarded. All unique reads without adapters in each sample were mapped to the 20 genomes using Bowtie 1.0.0 (28) (parameters: -a -v 0 -m 10) and only perfect matches over their entire length were kept. The leftmost (5′ end) nucleotide of the mapped reads represented the first nucleotide downstream of the cleavage positions, and reads with the same 5′ start position in the genome were summed and their counts were normalized by the total number of mapped reads to obtain the reads per million (RPM) values of the cleavage positions. Cleavage positions were annotated and assigned into one of following categories: CDS, 3′-UTR, exon, 5′-UTR, intron and intergenic region. Subsequently, cleavage positions with 20 (25 for piRNAs) extra bases upstream (5′) and 15 extra downstream (3′) were extracted as target-signature sequences (t-signatures) (19). Finally, t-signature sequences were used to construct the bowtie index using bowtie2-build software with default parameters (29).

### Evaluation of sRNA–target interactions using novel alignScore software

To predict the targets of sRNAs, we mapped sRNAs to t-signature sequences constructed with the aforementioned steps using Bowtie 2 (version 2.2.4) (29). After surveying the alignments of experimentally validated miRNA–target interactions (MTIs) in the reference set (Supplementary Table S1), we found that most of them (119 of 121) presented just one mismatch in a > = 10 nt stretch of a miRNA–target duplex. Considering the tradeoff between time and sensitivity, we provided two modes with the following search options in Bowtie 2:

- Stringent mode
  -a - -mm -N 1 -L 10 -i S,1,1 - -score-min L,-50,0 - -rdg 0,6 - -rfg 0,6 - -gbar 2 - -nofw - -non-deterministic - -no-unal - -no-sq
- Loose mode
  -a - -mm -N 1 -L 7 -i L,2,0.1 - -score-min L,-50,0 - -rdg 10,6 - -rfg 10,6 - -gbar 2 - -nofw - -non-deterministic - -no-unal - -no-sq

To predict targets and their cleavage sites, we developed a novel tool, alignScore (written in the C programming language), to evaluate alignments between sRNAs and targets using a proven scoring schema with some modifications and to determine if the cleavage sites of targets were at the sRNA positions 9, 10 and 11 in plants and animals (9,30–32). The default penalty score rule in the alignScore software is described as follows: (i) 0.5-point penalty for G:U wobbles in the non-core region (nt 14–21); (ii) 1-point penalty for G:U wobbles in the core region (nt 2–13), indels/mismatches in the non-core region (nt 14–21); (iii) 1-point penalty for sites lacking an A across from sRNA nt 1 and (iv) 2-point penalty for indels or mismatches in core region (nt 2–13). The input file of alignScore can be in the SAM or BAM format.

### Quantifying the abundance of degradome fragments using the degradomeBinomTest tool

The distribution of degradome fragments across the transcripts is assumed to be random or have no bias; therefore, the probability (*p*) of fragments being at a specific 1-nt position of a certain transcript equals 1/(*L*−*l*+1), where *L* and *l* are the lengths of the target transcript and degradome tag, respectively. Therefore, the probability of *k* or more fragments at an interested position followed a binomial distribution:

\begin{equation*} P(X \ge k) = \sum\limits_{x = k}^n {\left( {\begin{array}{*{20}c} n \\ x \\ \end{array}} \right)p^x (1 - p)^{n - x} ,\,p = \frac{1}{{L - l + 1}}} \end{equation*}

where *k* is the observed counts of tags assigned to a given position of the target transcript, which resides in the 9–11th nucleotide of the duplex from the sRNA 5′ end, and *n* is the total number of tags mapped to the target transcript. Here, *P* represents the probability which the target transcript tends to be cleaved by the sRNA in a particular position. A low *P*-value suggests high confidence of sRNA-mediated cleavage on the designated position of the transcript. We also developed a novel tool, degradomeBinomTest, which was written in the C programming language with the cdflib static library imported, to estimate the *P*-value of degradome fragments. All *P*-values were adjusted with the False Discovery Rate (FDR) correction (33).

### Classifying sRNA-mediated cleavage events

A sRNA-mediated cleavage event could be classified by its degradome tag abundance level across the target transcript. StarScan employed two 4-category systems that were similar to those defined in CleaveLand4 (34) and sPARTA (22), respectively, with some modifications. One was applied to annotated transcripts as follows: (i) Category Z is the case in which the tag abundance at a given position is equal to the maximum on the transcript and there is only one position at the maximum value; (ii) Category I is the case akin to Category Z, except that there is more than one position at the maximum value; (iii) Category II is the case in which the tag abundance at a given position is less than the maximum but larger than the average value on the transcript and (iv) Category III is the case in which the tag abundance at a given position is less than the average value on the transcript. The other was adapted to introns and unannotated regions as follows: (i) Category Z is the tag abundance at a given position above the 90th percentile of all tag abundances; (ii) Category I is the tag abundance at a given position below the 90th percentile but greater than the 75th percentile; (iii) Category II is the tag abundance at a given position below the 75th percentile but greater than the median and (iv) Category III is the tag abundance at a given position below the median.

## IMPLEMENTATION

The web server of StarScan was constructed under the Apache/PHP/MySQL environment in the Linux system. The back-end pipeline was implemented in Perl. The server runs on 64 bit CentOS release 5.9 with a 16-core 2.67 GHz Intel Xeon CPU and 32 GB RAM. The web application was implemented for multiple platforms and has been successfully tested in Chrome 40, Firefox 37, Opera 28, Safari 5, Internet Explorer 9–11. The workflow developed in this study was time-efficient, which took only 12 s for the smallest-sized sample (the Miwi mutant sample of mouse testes) and 3 min 42 s for the medium-sized sample (the Hela rep4 sample), both randomly used 100 sRNA sequences from miRBase version 21 and the loosest filtering criteria in the stringent mode.

## DATA INPUT

StarScan provides a simple, user-friendly interface that allows users to extensively scan small RNA targets in degradome sequencing data via a web server. The main input of StarScan includes nucleotide sequences from diverse sRNAs (miRNAs, siRNAs, piRNAs, etc.) in the FASTA format. Raw or formatted sequence data can be pasted directly into the query sequence box or uploaded from a local file. In addition, a number of important parameters are provided. Users can set the cutoff of RPM, Cleavage Site, Category, Penalty Score, FDR and Region types before submitting a job. After data submission, a general run (100 sRNA sequences as input) without error messages returned might take several minutes to finish when testing the medium-sized sample. Users can bookmark the result page or save the page link and retrieve their results from the link with a unique ID that is randomly generated by the server for each job. If users provide a valid email address, StarScan will send out a notification with the link.

## DATA OUTPUT

**Figure 2.**
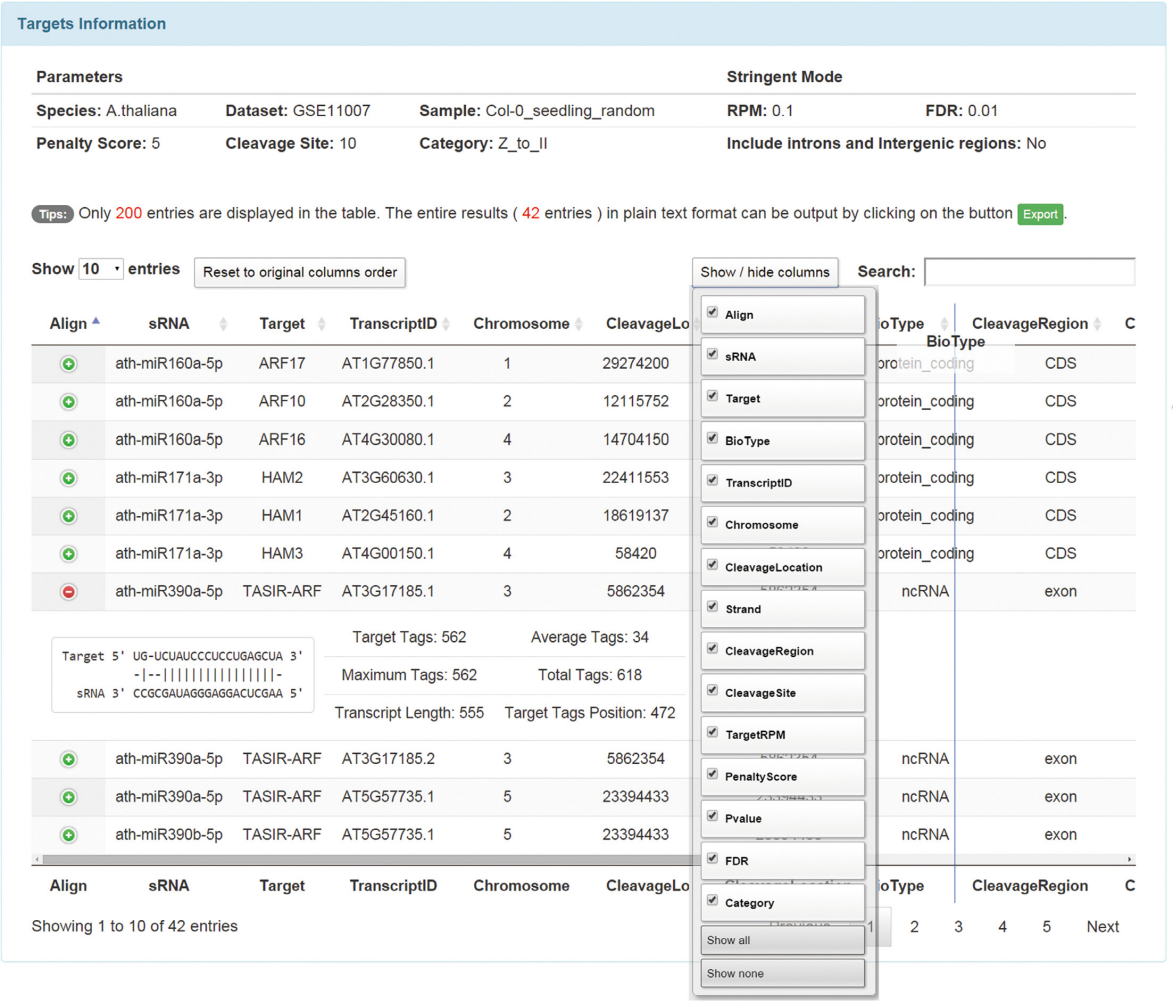
The results page of StarScan. This example used 10 *Arabidopsis* miRNAs to search against the seedling sample from GSE11007 with default parameters.

The results page (Figure 2) mainly consists of a data table that includes more than 20 distinct fields to describe the details of target hits. Detailed information such as the cleaved positions on the transcripts and corresponding coordinates on the genomes, summary of the degradome tags attribution on the individual transcripts and sRNA–target pairing quality is displayed succinctly and in a user-friendly format. The results include the cleavage positions on genomes and transcripts, cleavage regions (CDS, 3′ UTR, exon, 5′ UTR, intron, intergenic) on transcripts, sRNA and target gene names, transcript ID, gene types (e.g. protein coding or ncRNA), cleavage sites (9, 10 or 11), penalty scores, categories, RPM and alignment diagrams, *P*-values and FDR calculated by our degradomeBinomTest tool. Users can reorder, hide or show any columns in the result table. Thus, it is convenient for data view and comparison in the user-defined vision style. By clicking the image symbol (‘+’) in the leftmost end of every row, the alignment between the sRNA and target will be shown. Moreover, the keyword search is supported to scale down the results. Only 200 entries of target information are displayed in the table, and users can obtain all results in text format by clicking on the ‘export’ button.

### Prediction performance of StarScan in experimentally validated MTIs

To evaluate the performance of StarScan, we collected 102 and 101 *Arabidopsis thaliana* MTIs that were validated by 5′ RACE, northern blot, etc., from two recent studies (35,36), respectively. After removing repeated and suspicious MTIs that were inconsistent with the descriptions of the original papers, a total of 121 non-redundant experimentally validated MTIs (Supplementary Table S1) were used as benchmarks for the assessment of the predictions of StarScan. As a consequence, 110 (90.91%) were recaptured in at least one of the 17 *A. thaliana* sample data deposited at StarScan without any cutoff limit. Next, we examined the consensus prediction from a third party predictor named CleaveLand4 (34), the frequently used and most-cited tool for computational validation of plant MTIs using degradome sequencing data at present. As shown in Table 1, with two different parameter values, StarScan discovered more MTIs from the reference set in the majority of samples in the stringent mode and exhibited an ultrafast speed in scanning small RNA targets. In addition, we added a comparison between both tools by using the recommended parameters in StarScan. StarScan could predict more other interactions that did not belong to the reference set due to the different Bowtie mapping parameters (Supplementary Tables S1 and S2). In the case of multiple valid alignments, only one was randomly selected by CleaveLand4. The *P*-value and category generated by CleaveLand4 were random and dynamic. However, the *P*-value and category generated by StarScan were accurate and fixed. On the other hand, it was difficult to evaluate whether those other interactions were false predictions because there were no standard methods in identifying targets from degradome sequencing data at present. We further examined whether other interactions identified by StarScan were reported by other published papers and found many interactions were predicted by other tools (e.g. PAREsnip, SeqTar and sPARTA) or experimentally validated (Supplementary Tables S1 and S2). For example, the interaction between ath-miR157a-5p and SPL2 (AT5G43270) has been experimentally validated (37). Moreover, many interactions were also predicted from multiple degradome sequencing datasets (Supplementary Tables S1 and S2). Therefore, these tests indicated that the computational workflow of StarScan could identify high-confidence MTIs from degradeome sequencing data.

**Table 1.** The comparison of the hit numbers in 121 experimentally validated *Arabidopsis* MTIs between CleaveLand4 and StarScan

			Parameter values 1*		Parameter values 2**	
GSE accession	Tissue	Treatment	CleaveLand4	StarScan	CleaveLand4	StarScan
GSE11007	inflorescence	dT primer	30	31	17 (1)	16 (8)
	inflorescence	random primer	53	62	31 (3)	35 (6)
	seedling	random primer	55	56	27 (2)	31 (5)
GSE11094	inflorescence	control	89	91	66 (15)	76 (70)
	inflorescence	xrn4 mutant	96	100	70 (17)	82 (59)
GSE47121	flower bud	rep A	71	76	43 (8)	50 (26)
	flower bud	rep B	71	76	43 (9)	52 (22)
GSE52342	inflorescence	control	60	65	47 (12)	45 (20)
	inflorescence	ddm1-2 mutant	85	88	47 (12)	61 (49)
	inflorescence	ddm1-2 & rdr6-15 mutant	79	86	54 (13)	60 (33)
	inflorescence	rdr6-15 mutant	80	83	53 (14)	52 (26)
GSE55151	leaf (early senescence)	rep 1	86	88	55 (15)	72 (59)
	leaf (early senescence)	rep 2	74	79	50 (13)	58 (60)
	leaf (late senescence)	rep 1	38	38	22 (7)	18 (8)
	leaf (late senescence)	rep 2	46	46	29 (4)	27 (23)
	leaf (mature)		91	97	55 (24)	74 (98)
	leaf (young)		95	99	54 (28)	75 (107)
All	All		105	110	89 (60)	90 (215)

*: Default parameters (no *P*-value filtering and all categories reported) in CleaveLand4. **: Default parameters in StarScan. Detailed options of StarScan: FDR < 0.01, category = 0–2, cleavage site = 10, RPM > 0.1, penalty score < = 5, stringent mode, Bowtie-specific parameters ‘-a -v 0 -m 10’; Detailed options of CleaveLand4: *P* < 0.01, category = 0–2, cleavage site = 10, Bowtie-specific parameters ‘-v 1 - -best -k 1’. The numbers inside the parentheses represented the counts of other MTIs that did not belong to the reference set.

## CASE STUDY

**Figure 3.**
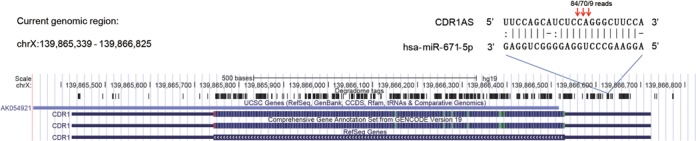
UCSC genome browser snapshot for displaying the cleavage positions of CDR1AS. Human hsa-miR-671-5p directed CDR1AS cleavage at the miRNA position 9 (9 reads), 10 (70 reads) and 11 (84 reads) with the corresponding hg19 genome coordinate chrX: 139 866 637–139 866 379 (+).

To illustrate that StarScan can be used to discover novel MTIs in unannotated genomic regions, all known human miRNAs (miRBase version 21) were submitted to our web server to scan their targets in brain samples from GSE22068 (parameters: stringent mode, 0.5 RPM, 2.5 penalty scores, all cleavage sites and categories reported). As a result, StarScan identified 29 MTIs that were located within unannotated regions and 116 interactions located within genic regions. The interactions located within unannotated regions had been missed in the original paper that generated these degradome sequencing data because they were limited to analysis of the annotated protein-coding regions. Surprisingly, the degradome tag abundance of three hsa-miR-671-5p targets located within intergenic regions (chrX: 139 866 637–139 866 379, sense strand, hg19) (Figure 3) was high and ranked first among all identified MTIs. Upon further literature and genomics analysis, we found that the region containing these three cleavage positions was still unannotated in Gencode V19, and even in the latest version, 21, but could be referred as the exon of circRNA CDR1AS (also known as ciRS-7) identified in a previous study (11). Interestingly, the three cleavage events identified by StarScan were also confirmed in a study using 5′ RACE experiments (11).

## DISCUSSION AND CONCLUSIONS

StarScan is a powerful web-based tool for discovering small RNA targets from degradome sequencing data. Compared to the existing programs for target screening which essentially focus on searching plant miRNA targets, StarScan provides an advanced search engine to identify cleavage targets of diverse classes of known and novel sRNAs in animals as well as plant species. Moreover, StarScan is the first tool that can be used to identify animal piRNA targets from degradome sequencing data. In addition, StarScan was designed to search for small RNA targets in multiple genomic regions, including the CDS, 3′ UTR, exons, 5′ UTR, introns and intergenic regions. The identification of interactions between hsa-miR-671-5p and circRNA CDR1AS, unannotated in the latest human genome version, demonstrated that StarScan can be used to discover novel regulatory modules. The strategy of scanning only the t-signature sequences to identify sRNA targets, which was introduced by StarScan, significantly reduced the size of the search space and the run-time compared to CleaveLand4. For instance, CleaveLand4 could be very time-consuming (around 14 h 56 min in Mode 2) when all 427 *Arabidopsis* sRNAs from miRBase were fed as input for the whole transcriptome analysis of the seedling sample from GSE11007, while StarScan finishes the analysis in ∼1 min 47 s. This speed allowed StarScan to analyze targets of hundreds of diverse RNAs at once. Although degradome sequencing identified tens of thousands of or even millions of cleavage sites in each of the tissues or cell lines, only hundreds of sites can be assigned to known sRNAs. Therefore, by integrating 100 degradome sequencing data from 20 species, StarScan will help researchers further investigate these data and discover novel regulatory modules and rules hidden in these data.

## SUPPLEMENTARY DATA

Supplementary Data are available at NAR Online.
